# Are Immigrants Scapegoats? The Reciprocal Relationships Between Subjective Well-Being, Political Distrust, and Anti-immigrant Attitudes in Young Adulthood

**DOI:** 10.1177/00332941211065951

**Published:** 2022-01-11

**Authors:** Liliia Korol, Alexander W. Fietzer, Pieter Bevelander, Ihor Pasichnyk

**Affiliations:** 1 Malmö Institute for Studies of Migration, Diversity and Welfare, 5264 Malmö University, Malmo, Sweden; 2 187502 National University of Ostroh Academy, Ostroh, Ukraine; 3 5924 Hunter College of the City University of New York, New York, NY, USA

**Keywords:** young adults, anti-immigrant attitudes, subjective well-being, political distrust

## Abstract

This study examined the impact of native youth’s subjective well-being on exclusionary attitudes toward immigrants, seeking to understand the relationship between subjective well-being, political distrust, and anti-immigrant attitudes over time. Using longitudinal data, we followed three cohorts of native young adults (*N* = 1352; *Mage* = 22.72, *SD* = 3.1) in Sweden over a period of 2 years. The results showed that subjective well-being did not predict an increase in anti-immigrant attitudes among native youth, but anti-immigrant attitudes had a significant impact on subjective well-being. The data also found bidirectional and mutually reinforcing relationships between subjective well-being and political distrust, and between political distrust and anti-immigrant attitudes. These results highlight that improving young adults’ subjective well-being represents an important basis for preventing the development of political distrust, which in turn could reduce native youth’s susceptibility to adopt hostile attitudes toward immigrants.

Increased migration due to globalization is changing the demography of many European countries, increasing their cultural, ethnic, and religious diversity. According to Eurostat data (2019), the number of foreign-born people in the European Union has continued to grow in recent years, reaching 39.9 million on January 2, 2018 (compared to 32.2 million in 2010). At the same time, xenophobia and anti-immigrant sentiments are growing among the EU native population (see, for example, Bevelander & Wodak, 2019), and in recent elections in European countries nationalist parties gained much of their electoral support in policies that framed immigration as an economic, cultural, and social threat. Such anti-immigrant attitudes jeopardize the harmonious integration of newcomers and risk deleterious consequences for the well-being, cohesion, and future prospects of the immigrant-receiving countries. Taken together, this points to the need to better understand factors that affect anti-immigrant attitudes, particularly among native young adults who shape the Europe of tomorrow.

## Hostility and Negative Attitudes toward Immigrants

Existing research on individual-level factors that shape attitudes toward immigrants has developed in two major directions (for a review, see, Ceobanu & Escandell, 2010; Hainmueller & Hopkins, 2014; Hodson & Dhont, 2015). The first line of research is built on rational-based group and competition theories (Schneider, 2008) and emphasizes the role of socio-economic factors. It claims that natives’ economic self-interest and concerns about the increase of low-skilled labor leading to lowered wages (or employment) may lead to anti-immigrant hostility (e.g., Billiet et al., 2014; Hayes & Dowds, 2006). Prior studies have shown that individuals who are economically vulnerable (e.g., unemployed, low educated, and unskilled/low-skilled workers) tend to express more negative views of immigrants than those who have a more secure socio-economic position (e.g., Hainmueller & Hiscox 2007; Semyonov & Glikman, 2009; Schneider, 2008).

The second line of research focuses on socio-cultural (e.g., McLaren, 2003; McLaren & Johnson, 2007; Sides & Citrin, 2007) and ideological (e.g., Cohrs & Stelzl, 2010; Costello & Hodson, 2011; Kupper et al., 2010) factors. One strand of this research is built upon the premise of social identity theory (Hogg, 2006) that individuals have a fundamental need to identify themselves with similar others and tend to perceive their ingroup as superior to outgroups. When natives perceive differences between the majority and minority groups as undermining their cultural majority position and challenging their national distinctiveness, they are more likely to hold hostile attitudes toward newcomers (McLaren, 2003; McLaren & Johnson, 2007; Sides & Citrin, 2007). Another strand of research (Duckitt & Sibley, 2017) argues that ideological orientations such as social dominance orientation (i.e., a personal preference for hierarchical and unequal intergroup relations) and right-wing authoritarianism (i.e., a predisposition toward social conformity to ingroup norms and valuing authority) predict prejudicial attitudes toward groups that are perceived as threatening and inferior, including those of immigrant background. Consistent with this theoretical perspective, prior research has provided empirical evidence of the strong associations between these two ideological orientations and anti-immigrant attitudes (for a meta-analysis, see Cohrs & Stelzl, 2010; see also Costello & Hodson, 2011; Kupper et al., 2010).

While prior research has primarily sought to explain negative attitudes toward immigrants through the perspective of a ‘threat paradigm’—that is, antipathy and antagonism toward immigrants are primarily driven by perceptions of threat that immigrants represent to an individual’s well-being—much less emphasis has been placed on how subjective well-being might affect this process. One would expect subjective well-being and perceived threats to individual economic or cultural well-being to be closely linked. Existing literature, however, argues that subjective well-being creates a psychological environment that regulates perceived threats to one’s life, whether actual or potential (Shrira et al., 2011; Shmotkin, 2005). That is, subjective well-being makes actual or potential adversity more manageable by filtering potential threats, thereby letting individuals evaluate their life situations as basically favorable (Shrira et al., 2011). Relatedly, the revised adaptation theory (Diener et al., 2018; Diener et al., 2006) proposes that negative events or adverse life circumstances might influence people’s immediate happiness; but by adapting to these changing circumstances, people tend to quickly return to their baseline levels of subjective well-being. Taken together, existing theoretical frameworks suggest that perceptions of actual or potential threats to one’s economic or cultural well-being might not necessarily equate to low subjective well-being. Thus, it is important to better understand whether subjective well-being has any role to play in affecting anti-immigrant attitudes.

We are aware of only two studies that have specifically attempted to investigate the link between subjective well-being and attitudes toward immigrants; they yielded inconsistent findings. Specifically, Sirgy et al., (2018) showed that individuals (i.e., Germans between the ages of 18 and 70) who experienced high levels of subjective ill-being (i.e., low levels of subjective well-being) were likely to be more Islamophobic. In contrast, Gordon (2018) found that life satisfaction (measured using the Personal Wellbeing Index) was not related to pro-immigrant sentiments among a nationally representative probability sample in South Africa (aged 16 and over). In addition, prior studies that included life satisfaction in their analyses as a control variable (e.g., Lancee & Sarrasin, 2015; McLaren, 2003) or as a proxy indicator for personal threat (McLaren, 2003) or social alienation (Sides & Citrin, 2007) reported weak or insignificant association with exclusionary feelings toward immigrants across countries. Hence, the empirical evidence on the role of individual subjective well-being in shaping anti-immigrant attitudes is unclear.

In addition, despite young adulthood being a particularly fertile developmental period during which to examine an individual’s prejudice as well as their feelings about the current state of their life trajectory, longitudinal research on young adults is scarce. To address this gap in our current knowledge, the present study aims to (1) investigate the longitudinal relationship between subjective well-being and anti-immigrant attitudes among native majority young adults, and (2) explore any relationship that might exist between subjective well-being, political distrust, and anti-immigrant attitudes.

## Subjective Well-Being and Anti-immigrant Attitudes

The existing literature proposes some theoretical arguments to explain a potential link between low subjective well-being of individuals and negative attitudes toward immigrants. For instance, as postulated by the frustration–aggression hypothesis (Berkowitz, 1989), aversive life circumstances (e.g., insecure life situation) may evoke stress and negative affect, causing people to react to such stressors or frustration with hostility and aggressive cognitions. When a person cannot aggress directly, they may instead displace their aggression toward an innocent target (Marcus-Newhall et al., 2000; see also scapegoat theory, Allport, 1954). Developing this line of reasoning further, a theory of triggered displaced aggression (Miller et al., 2003; Pedersen et al., 2000) posits that the displacement target often provides a trivial second provocation (i.e., a trigger) that in turn can produce disjunctively escalated aggressive responding. Thus, aggression resulting from a triggering event is incommensurate with its level of provocation and can disjunctively exceed the independent additive effects of the initial provocation and the trigger. Applied to the context of the present study, native individuals who are unable to achieve their life goals and fulfill their needs are likely to feel stressed, frustrated, and hostile. Immigrants who are portrayed by mass media and political discourse as exacerbating social problems and drawing on public welfare resources may then provide the negative emotional trigger. Hence, natives may direct their escalated aggressive responding at immigrant-origin individuals and adopt xenophobic attitudes. Alternatively, Reijntjes et al. (2013) argue that ethnic minority outgroups (i.e., immigrants) may be especially likely to be displacement targets due to perceived dissimilarity and negativity associated with outgroup status.

Another possible explanation relies upon the conceptual premises of uncertainty-identity (Hogg, 2014) and social threat (Agroskin & Jonas, 2010; Aydin et al., 2014; Fritsche et al., 2011) theories. As argued by (Yoxon et al., 2017), perceiving oneself as relatively deprived (i.e., concerning other group members or their own expectations) is likely to result in feelings of uncertainty about one’s status in the society. This in turn makes individuals more prone to seek ways to regain control over their lives, particularly by adopting ethnocentric views and xenophobic attitudes toward minorities (Aydin et al., 2014; Fritsche et al., 2011). Supporting these theoretical arguments, recent empirical research (Yoxon et al., 2017) based on nationally representative samples in nine European countries showed that individuals who perceived themselves as relatively deprived were also more likely to hold prejudicial attitudes toward immigrants. These theoretical perspectives suggest that by evoking feelings of fear, uncertainty, or frustration, poor subjective well-being may make native individuals more likely to adopt anti-immigrant attitudes as a means to cope with personal disappointment or to restore the sense of control of their lives.

At the same time, there is evidence that the direction of effect is the reversed; anti-immigrant attitudes lead to poor subjective well-being. For instance, Dinh et al., (2014) found that prejudicial attitudes (e.g., racism, homophobia, and anti-immigrant sentiment) were negatively associated with psychological, social, and physical well-being among college students in the US. Relatedly, a recent study based on European Social Surveys from 2002 and 2014 comprising 44,721 individuals living in 18 countries (Bazán-Monasterio et al., 2021) showed that rejection of the arrival of immigrants had a strong negative influence on life satisfaction. In addition, the study demonstrated that this effect was stronger for people from Generation Y (born between 1981 and 1997) than for the older generation. Thus, to examine these competing hypotheses, we investigated the reciprocal relationship between subjective well-being and anti-immigrant attitudes among native young adults over time.

## The Potential Role of Political Distrust

Subjective well-being may have an important role to play not only in shaping young adults’ attitudes toward immigrants but also in affecting their attitudes toward broader society. Recent research provides evidence that subjective well-being is closely linked to individuals’ satisfaction with democracy (Hooghe, 2012), political support (Esaiasson et al., 2019), and political trust (Mattila & Rapeli, 2018). For instance, Hooghe (2012) found that Belgian citizens (aged 18 and over) who reported higher levels of subjective well-being were more likely to be satisfied with the way democracy worked in Belgium and with the type of society they lived in. Similarly, (Esaiasson et al., 2019) showed that subjective well-being was strongly related to political attitudes (e.g., democratic satisfaction and tax evasion) across European countries, even controlling for confounding factors at country and individual level. Furthermore, the researchers found that people who had experienced a critical life event (i.e., the termination of a close personal relationship) were less supportive of government, and that this relationship was mediated by changes in life satisfaction. Relatedly, in research based on European Social Survey data in 19 West European countries from 2002–2012, Mattila and Rapeli (2018) found that citizens aged 15 and over who reported poor subjective health were more likely to have reduced levels of trust in important political institutions (e.g., parliament, politicians, and political parties).

Why might individuals with poor subjective well-being be more prone to display political distrust? One possibility is to view political trust as a contractual relationship between individuals and the political system (i.e., governmental institutions and political actors). As postulated by psychological contract theory, contractual relationships function as an analytical tool that links citizens and their schemas (i.e., attitudes and beliefs) to the larger social structures (for further discussion see Rubin, 2012). This theoretical perspective assumes that individuals extend their support to a political system only when they feel that they are receiving certain benefits from this system (e.g., security and reasonable level of financial well-being) and life conditions that meet their expectations. Deviations from these expectations (such as deterioration of living conditions or poor subjective well-being) are seen as breaches of this contract that, in turn, might lead to reduced trust and confidence in the political system (e.g., the happiness contract model, Esaiasson et al., 2019; the psychological-democratic trust contract, Mattila & Rapeli, 2018). Thus, when young adults experience poor subjective well-being, they might blame the political system for their hardships and reciprocate by displaying distrust and lack of confidence in the performance of the whole system, including its institutions and actors.

At the same time, there is evidence that people who exhibit distrust and dissatisfaction with the political system in their country tend to hold anti-immigrant attitudes. For instance, Ford and Goodwin (2014) found that those who oppose the EU and European integration are more likely to hold anti-immigrant attitudes. Jiang and Ma (2020) identified political distrust as the most important attitudinal factor underlying support of far-right parties, which often center their policies around anti-immigrant rhetoric (see Ceobanu & Escandell, 2010). A large-scale youth survey in 14 European countries (Mieriņa & Koroļeva, 2015) showed that xenophobia, welfare chauvinism, and exclusionary attitudes toward immigrants were more widespread among young adults (aged 16–25) who had little interest and understanding of politics and low political trust. Aschauer, 2016, based on the 2006 and 2012 waves of the European Social Survey, found that individuals who reported higher levels of political disenchantment (e.g., political distrust, dissatisfaction with the national government, and the way democracy works in the country) were more likely to perceive immigrants as a threat, especially in Western Europe. Even though prior research has established positive linkages between subjective well-being and political distrust, on the one hand, as well as political distrust and anti-immigrant attitudes, on the other hand, to our knowledge no study has attempted to examine their potential cross-lagged relationships. Examining these potential reciprocal relationships is important for theory and practice, particularly intervention programs directed at reducing youth’s susceptibility to adopt exclusionary attitudes toward immigrant-origin individuals. The present study aimed to address this important gap in current knowledge.

### The Present Study

Experiencing positive well-being and satisfaction with life are important developmental tasks during young adulthood (Arnett, 2015), particularly for the present generation of young adults in Western societies (Mayseless & Karen, 2014). Moreover, prior research (Henry & Sears, 2009) provides evidence that prejudicial attitudes continue to crystallize as adulthood progresses. Thus, in the present study, we chose to focus on young adults and aimed to extend our understanding of the relationship between subjective well-being and anti-immigrant attitudes during this important developmental period. Specifically, we addressed two theoretically important questions:

#### Research question 1: Does subjective well-being impact anti-immigrant attitudes among young adults?

Based on recent empirical findings in the field (Sirgy et al., 2018; Yoxon et al., 2017), we hypothesized that low subjective well-being would make young adults more likely to adopt exclusionary attitudes toward immigrants over time (*Hypothesis 1a*). In addition, given prior empirical evidence on the positive association between prejudicial attitudes and young adults’ well-being (Bazán-Monasterio et al., 2021; Dinh et al., 2014) and on more speculative basis, we anticipated that the direction of the suggested effect may be the opposite, namely, that anti-immigrant attitudes would lead to low subjective well-being (*Hypothesis 1b*).

#### Research question 2: Do subjective well-being, political distrust, and anti-immigrant attitudes among native young adults reciprocally influence each other?

**Figure 1.**
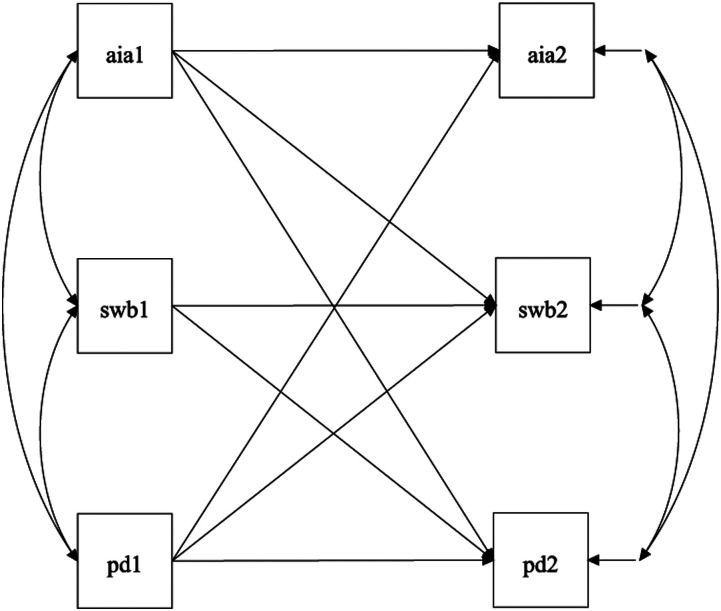
Hypothesized cross-lagged panel model of subjective well-being (SWB), political distrust (PD), and anti-immigrant attitudes (AIA). Numbers after variables indicate the wave at time at data collection, with T1 = Time 1 and T2 = Time 2. All hypothesized paths are shown.

Given the paucity of prior longitudinal research in this direction and given the potential competing but not mutually exclusive causes already found in the literature, we expected that there might be a reciprocal relationship between subjective well-being and political distrust (*Hypothesis 2a*) as well as political distrust and anti-immigrant attitudes (*Hypothesis 2b*) across time. Figure 1 displays the resulting hypothesized cross-lagged panel model.

Finally, prior research suggests that individuals with lower perceived socio-economic status (SES) often report lower subjective well-being (e.g., Navarro-Carrillo et al., 2020; for a meta-analytic review, see also Tan et al., 2020) and show more severe health issues (see, for example, Cundiff & Matthews, 2017 for a meta-analysis). In addition, recent studies provide some evidence that individuals who report low perceived SES are more likely to display higher levels of political distrust (Haugsgjerd & Kumlin, 2020) and more negative attitudes toward immigrants (Heizmann & Huth, 2021). Therefore, we controlled for perceived SES in all analyses. To examine these research questions, we used a longitudinal design that followed three cohorts of native majority young adults (i.e., 20-, 22-, and 26-year-olds) over a period of 2 years.

## Method

### Participants

The data for the present study were extracted from a large longitudinal survey collected for the Political Socialization Program (Amnå et al., 2009) and conducted in a city of about 137,000 inhabitants in Sweden. The city is similar to the national average on population density, unemployment, income level, and share of immigrant population (Centralbyrån, 2019). Our sample consisted of three cohorts of young adults (20-, 22-, and 26-year-olds) who were followed twice with a time lag of 2 years between data collections. The target sample comprised approximately 1000 individuals in each age group, who were randomly selected from a list of all 20–26 year-olds living in the city, provided by the county. The questionnaires were mailed to the target sample, together with a personalized link to an online version of the questionnaire. Participants received a gift card of 28€ for taking part in the survey. All the procedures were approved by the Regional Ethics Board in Uppsala.

Only young adults who met the criterion of native origin were included in the analyses. We excluded individuals with at least one parent born outside of Sweden. After processing the data, the final analytic sample comprised 1352 individuals (i.e., 475 20-year-olds, 416 22-year-olds, and 468 26-year-olds). The sample consisted of 57.3% young females. Regarding education level, 20.7% of the respondents finished vocational high school, 40.7% finished academic high school, and 27.8% finished university college or university. A majority of young adults reported they had moved away from home and had no children (80.6% and 91.4%, respectively). In response to a question about their civil status, 36.9% stated that they were married/co-habiting, 19.7% mentioned that they had a partner/partner is a separate residence, and 43.1% reported that they were single.

### Measures

#### Subjective well-being

To measure subjective well-being, we focused on the cognitive dimension of subjective well-being and assessed perceived life situation. This measure was created specifically for this survey. Respondents were presented with a stem statement (“My life situation right now”), and then they were asked to evaluate the following six items: “I don’t have any chance to come out onto the labor market today, I am in an insecure work situation, or I am unemployed”; “My education is not sufficient for me to do what I would like to do”; “Due to an insecure life situation, I can’t afford to do the things that others of my age do”; “Today, I see no possibility of getting the home I would like to have”; “Due to an insecure life situation, I find it hard to establish the friendships that others have”; “It seems that I don’t have any opportunities to influence my own life situation right now.” Responses were given on a four-point Likert-type scale, ranging from 1 (*does not apply at all*) to 4 (*applies very well*). Higher scores indicated more negative evaluation of a youth’s current life situation (Cronbach’s α_T1_ = .77, α_T2_ = .78).

#### Political distrust

Political distrust was measured via two items concerning youths’ degree of confidence in the parliament and the government taken from a seven-item scale on political institutions (Linde, 2004; Norris, 1999). Responses were given on a 4-point Likert-type scale, ranging from 1 (*a lot of trust*) to 4 (*no trust at all*). Higher scores indicated greater degree of distrust toward political institutions (Cronbach’s α_T1_ = .80, α_T2_ = .81).

#### Anti-immigrant attitudes

Attitudes toward immigrants were assessed via five items adapted from prior research on inter-group attitudes (van Zalk et al., 2013; van Zalk & Kerr, 2014). Respondents were presented with the stem question “What are your views on people who have moved here from other countries?” and were asked to rate items on a four-point Likert-type scale, ranging from 1 (*does not apply at all*) to 4 (*applies very well*). Sample items included: “Immigrants often come here to take advantage of the welfare in Sweden” and “Immigrants often take jobs from people who are born in Sweden.” Previous studies (van Zalk et al., 2013; van Zalk & Kerr, 2014) provided evidence on the convergent, predictive, and discriminant validity of the scale. Higher scores indicated greater anti-immigrant attitudes. The scale demonstrated strong inter-item reliability (Cronbach’s α_T1_ = .85, α_T2_ = .86).

#### Control variables

In the present study, we controlled for gender (1 = female) and socio-economic status (SES).

##### Perceived socio-economic status

To assess how young adults perceived their socio-economic status, they were asked if it happened that they had had difficulty in handling their ongoing expenses (for food, rent, other household items, etc.; 1 = yes, 0 = no).

## Results

### Preliminary Analyses

The initial dataset contained 1359 participants who had responded to some portion of the survey. Of these, seven respondents were deleted for missing data on key variables at Time 1 (T1) or Time 2 (T2), for a total of 1352 participants. We conducted an attrition analysis by regressing the three T1 variables along with gender and SES onto a dummy dropout variable (where 0 = dropout, 1 = retention). Results of the analysis were statistically significant (likely due to the large sample size) but only explained 3.4% of the variance in dropout χ^2^ (20) = 44.46, *p* = .001, *R*^*2*^_*McFadden’s*_ = .034. Because of the large number of predictor variables (20), we set the probability of making a Type I error at .01. None of the T1 predictor variables were significant at the .01 level. However, gender (*z* = −4.14, *p* < .001) was statistically significant, with a larger number of males (*n* = 166) dropping out of the study than females (*n* = 131). As a result, we also examined the impact that gender had on the model.

All data analyses were conducted using Stata 14.2 and Mplus (Version 7.3, (Muthén & Muthén, 1998). Table 1 lists the descriptive statistics and correlations between variables in the study. Across the three variables at T1, missing values ranged from 0.0 to 1.2%. Across the three variables at T2, missing data ranged from 21.5 to 21.8%. Imputation of missing data was carried out by the Mplus program. Analysis of normality for the six variables found statistically significant violations of skewness in all six variables, and five variables were significantly kurtotic. To correct for non-normality of data and potential non-independence of observations, we ran the main analysis using the Yuan-Bentler T2* (Yuan & Bentler, 2000) estimation method (MLR in Mplus).

**Table 1.** Descriptive Statistics, Pairwise Correlations, and Coefficient Alphas of Subjective Well-Being, Political Distrust, and Attitudes toward Immigrants.

	*N* ^a^	Range	*M (SD)*	Skew	Kurtosis	Pearson correlations^bc^					
						AIA1	SWB1	PD1	AIA2	SWB2	PD2
T1 anti-immigrant attitudes	1345	1–4.0	1.82 (.66)	.80	.32	.85					
T1 subjective well-being	1347	1–4.0	1.70 (.61)	1.00	.55	.16	.77				
T1 political distrust	1336	1–4.0	2.33 (.51)	.58	.93	.29	.26	.80			
T2 anti-immigrant attitudes	1056	1–4.0	1.82 (.67)	.78	.17	.79	.13	.27	.86		
T2 subjective well-being	1061	1–4.0	1.64 (.71)	1.09	.80	.18	.62	.24	.16	.78	
T2 political distrust	1056	1–4.0	2.41 (.52)	.41	.58	.26	.30	.64	.27	.26	.81

*Note.* T1 = Time 1. T2 = Time 2. AIA1 = anti-immigrant attitudes at T1. SWB1 = subjective well-being at T1. PD1 = political distrust at T1. AIA2 = anti-immigrant attitudes at T2. SWB2 = subjective well-being at T2. PD2 = political distrust at T2.

^a^Total *N* for the study was 1352.

^b^All correlations were significant at *p* < .001.

^c^The numbers on the diagonal in bold are coefficient alpha for the sample of each variable.

### Cross-Lagged Panel Model

**Figure 2.**
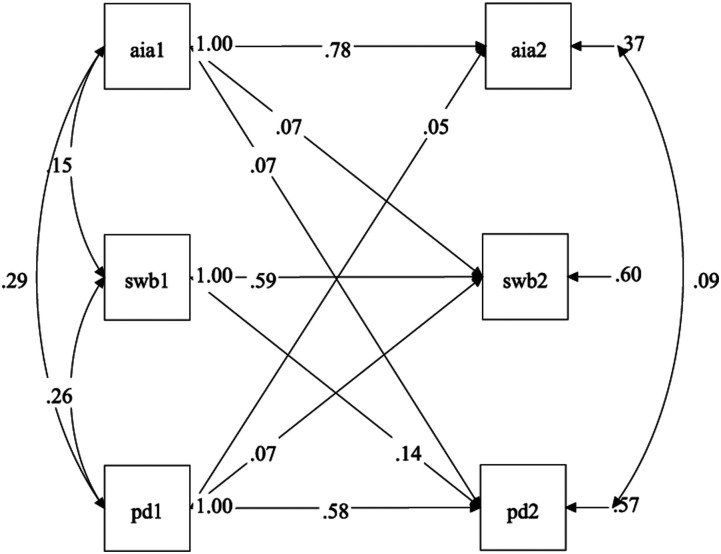
Observed cross-lagged panel model of subjective well-being (SWB), political distrust (PD), and anti-immigrant attitudes (AIA). Numbers after variables indicate the wave at time at data collection, with T1 = Time 1 and T2 = Time 2. Only statistically significant paths are shown.

We used cross-lagged panel modeling to examine the relationship between subjective well-being, anti-immigrant attitudes, and political distrust across time. Figure 2 displays the results of the analysis, and Table 2 lists the standardized regression coefficients between variables at each time point. As would be expected, the highest regressions are between the same variables at each time point (e.g., anti-immigrant attitudes at T1 and T2). These range from .62 to .79. There was no statistically significant effect of subjective well-being at T1 on anti-immigrant attitudes at T2, but there was a significant effect of anti-immigrant attitudes at T1 on subjective well-being at T2 (*β* = .05, *p* = .021). At the same time, there was a reciprocal, statistically significant effect between subjective well-being and political distrust. Specifically, subjective well-being was more predictive of political distrust (*β* = .14, *p* < .001) than political distrust was of subjective well-being (*β* = .07, *p* = .011). Further, there was a reciprocal, statistically significant effect between anti-immigrant attitudes and political distrust. Specifically, political distrust at T1 predicted anti-immigrant attitudes at T2 (*β* = .05, *p* = .022), while anti-immigrant attitudes at T1 predicted political distrust at T2 (*β* = .07, *p* = .015).

**Table 2.** Standardized Structural Regression Coefficients and 95% Confidence Intervals for Direct Effects of the Cross-Lagged Panel Model.

	Direct effect (95% CI)
Anti-immigrant attitudes at T2 on	
PD1	.05 (.01–.09)*
AIA1	.78 (.75–.81)***
SWB1	.00 (−.04–.04)
Political distrust at T2 on	
PD1	.58 (.53–.63)***
AIA1	.07 (.01–.13)**
SWB1	.14 (.08–.19)***
Subjective well-being at T2 on	
PD1	.07 (.02–.13)*
AIA1	.07 (.01–.13)*
SWB1	.59 (.54–.64)***

*Note.* CI = confidence interval. PD1 = political distrust at T1.

AIA1 = anti-immigrant attitudes at T1. SWB1 = subjective well-being at T1.

*p < .05.

** *p* < .01.

***p < .001

We also examined differences on the T1 variables for gender. There was a statistically significant difference on anti-immigrant attitudes at T1 by gender *t* (1317) = **−**6.87, *p* < .001, with males reporting higher anti-immigrant attitudes (*M* = 2.00, *SD* = .70) than females (*M* = 1.75, *SD* = .62). This carried over to T2 *t* (1039) = −5.64, *p* < .001, with males again reporting higher anti-immigrant attitudes (*M* = 1.96, *SD* = .71) than females (*M* = 1.72, *SD* = .63). However, when we added gender as a time-invariant predictor to the cross-lagged panel model the results were identical in terms of statistically significant paths. As a result, we omitted gender from the final model for parsimony.

Finally, we added cohort (a proxy for age) as a time-invariant predictor to all variables in the model. The result was the same pattern of significant predictors paths variables as in the cross-lagged panel model without cohort as a time-invariant predictor. Cohort predicted subjective well-being at T2 (*β* = **−**.07, *p* < .001), subjective well-being T1 (*β* = −.17, *p* < .001) and anti-immigrant attitudes (*β* = −.11, *p* < .001) at T1. Thus, for the current sample age does not significantly predict political distrust or anti-immigrant attitudes over time, though younger age is predictive of higher anti-immigrant attitudes and lower subjective well-being. Age does predict subjective well-being over time, with younger age predictive of higher subjective well-being. As with gender, we omitted cohort from the final model to be more parsimonious.

## Discussion

Prior research has largely focused on explaining anti-immigrant attitudes by examining natives’ perceptions of threats posed by immigrants to their economic (e.g., Hainmueller & Hiscox 2007; Semyonov & Glikman, 2009; Schneider, 2008) and cultural well-being (e.g., McLaren, 2003; McLaren & Johnson, 2007; Sides & Citrin, 2007). Less attention, however, has been paid to understanding the relationship between subjective well-being and anti-immigrant hostility (Gordon, 2018; Sirgy et al., 2018). To address this, the present study had a two-fold aim. First, we aimed to investigate whether low subjective well-being would generate anti-immigrant attitudes (or vice versa) among native youth in Sweden over time. Second, we aimed to explore reciprocal relationships between subjective well-being, political distrust, and anti-immigrant attitudes.

Consistent with prior cross-sectional research (e.g., Gordon, 2018; Sirgy et al., 2018), the data revealed that low subjective well-being was positively associated with anti-immigrant attitudes at both times of measurement. Although cross-sectional findings confirmed our hypothesis about the link between subjective well-being and anti-immigrant attitudes, the cross-lagged panel modeling showed no evidence of significant over-time impact of subjective well-being on anti-immigrant attitudes among Swedish young adults. At the same time, the results revealed that anti-immigrant attitudes had a positive effect on low subjective well-being. This finding could imply that holding negative attitudes toward immigrants may result in adverse cognitive consequences (or psychological costs; see, for similar reasoning, Kivel, 1996; Spanierman & Heppner, 2004) for native youth such as negative evaluations of their life situation. As it has been theorized by existing literature on racism, exclusionary attitudes toward members of different racial (or ethnic) groups tend to harm those who perpetuate them (Spanierman & Poteat, 2005) and can negatively impact a perpetrator’s sense of self-worth and their self-esteem (Kivel, 1996). As a result, individuals may place little value on themselves and their life achievements, feel they are worth less than others, and, thus, experience poor subjective well-being. Future research should replicate and extend the findings reported in the current study by using a more extended cross-lagged panel model that comprises three or more waves of data.

The present study goes beyond prior research by demonstrating that the relationship between native youth’s subjective well-being and political distrust is bidirectional. As postulated by contract theory (Esaiasson et al., 2019; Mattila & Rapeli, 2018), native young adults with low subjective well-being probably hold governmental institutions accountable for their unsatisfactory level of current life trajectory and insecure life situation and respond by expressing distrust and discontent with their performance. At the same time, as demonstrated by our findings, this relationship is interdependent, although asymmetric. That is, feelings of political disenchantment in turn make native young adults more likely to experience low subjective well-being over time. Although more longitudinal evidence is needed, this study indicates that the relationship between poor individual well-being and perceptions of broader society (e.g., political system and government) has a downward spiral pattern: poor subjective well-being triggers political discontent, which in turn leads to individual life insecurity.

A noteworthy finding of the present study is that there is a reciprocal relationship between native youth’s political distrust and anti-immigrant attitudes over time. Specifically, political distrust not only fuels exclusionary attitudes toward immigrants among native majority young adults, but anti-immigrant attitudes also generate political distrust. It is possible that political disenchantment may induce fears of social decline among native youth; consequently, they may embrace ethnocentric and exclusionary ideologies as a way to enhance cohesion and strengthen bonds of their ingroup. In line with this reasoning, a recent study by Aschauer & Mayerl, 2019, employing data from the European Social Survey across 21 countries, found that people who experienced high levels of social malaise (i.e., conceptualized as a cluster of dissatisfaction with society, political distrust, fear of societal decline, lack of recognition, and social distrust) were more likely to report higher levels of perceived ethnic threat from immigrants. At the same time, it is also possible that native youth who adopt negative attitudes toward immigrants may become more prone to support anti-immigrant and right-wing parties (which often build up their ideology on anti-immigrant rhetoric), which in turn may fulminate their discontent of the mainstream parties and the established political elite. Indeed, prior research has shown that voters of right-wing populist parties tend to be dissatisfied with the way democracy works in their country (e.g., Bowler et al., 2017; Pauwels, 2014). Further research is needed to determine the validity of these alternative explanations and extend the findings reported here.

### Strengths, Limitations, and Future Directions

The present study has several strengths. In particular, it provides evidence that the relationship between subjective well-being and political distrust, on the one hand, and political distrust and anti-immigrant attitudes, on the other, is reciprocal. Thus far, no longitudinal research has been conducted to examine the bidirectionality of the relationship between subjective well-being, political distrust, and anti-immigrant attitudes among native majority youth. The relationship between poor subjective well-being and political distrust among young adults of native background is mutually reinforcing: poor subjective well-being leads to political disenchantment over time, which in turn fosters further insecurity of important life domains. This study is the first to show that native youth’s political distrust not only generates exclusionary attitudes toward immigrants, but that adopting anti-immigrant attitudes by these youths also fuels feelings of political distrust. Moreover, the present study proposed and tested a novel view that native youth with poor subjective well-being are more prone to adopt anti-immigrant attitudes over time. Even though our data do not provide empirical evidence for this link, our study suggests that this relationship may develop the other way round. Specifically, the findings demonstrate that anti-immigrant attitudes have a positive effect on low subjective well-being in the long run. Finally, unlike the majority of existing studies in the field, this study focuses on young adulthood and covers three different age cohorts of young adults (i.e., 20-, 22-, and 26-year-olds). By doing so, it extended our understanding of the relationship between personal and political factors with anti-immigrant attitudes beyond the college years and through young adulthood when outgroup attitudes continue to crystalize (Henry & Sears, 2009).

Several limitations must be acknowledged when interpreting the findings of the current study. First, we did not include cultural or economic threats as control variables in our analyses, which was due to the design of the data set used in the present study. Therefore, we were not able to assess the independent influence of subjective well-being and political distrust on young adults’ anti-immigrant attitudes, above and beyond the contribution of threat perceptions. As such, future longitudinal research with additional measures of threat is needed to examine the unique role of individual and political factors in shaping anti-immigrant attitudes among native youth. Moreover, future studies may also seek to include a subjective measure of SES (e.g., MacArthur Scale of Subjective Social Status; Adler et al., 2000) and analyze its potential role in affecting young adults’ attitudes toward immigrants^1^. For instance, it is possible that young adults’ subjective assessments of their socio-economic standing relative to others in society (i.e., subjective SES) might affect their subjective well-being, which in turn might result in their adoption of anti-immigrant attitudes. Thus, future longitudinal research with a more comprehensive set of variables will provide a more complete understanding of the role of subjective well-being in shaping anti-immigrant attitudes in young adulthood.

Second, the measures used in this study were self-reported, which could be susceptible to common method or social desirability biases. Future research may eliminate this possibility by supplementing the self-reported measures with other informant-reports (e.g., from family members or friends). This would also open up the opportunity to replicate and confirm the current findings by applying different measures of subjective well-being, political distrust, and anti-immigrant attitudes, and thus advance the research in this direction. Third, we tested the hypothesized relationships among native young adults from a single Swedish city. This city is similar to the country as a whole with regard to its share of immigrant population, income, level of education, population density, and unemployment rate. Yet, whether the current findings are valid for young adults in other countries is an open question. Youth’s experiences of subjective well-being are embedded in specific socio-cultural contexts, which not only differ in the patterning and content of well-being, but also in its causes and outcomes (Tov & Diener, 2009). For instance, prior research provides evidence that life satisfaction varies among nations that differ in economic development (e.g., Diener et al., 2010), and European cultures have higher levels of subjective well-being than Pacific Rim cultures (e.g., Diener & Oishi, 2004; Tov & Diener, 2009). Therefore, the findings reported here need to be cross-validated in other countries. Finally, this study focused on the role of subjective well-being and political distrust in shaping negative outgroup attitudes among native youth. Future research may contribute to the literature by examining whether the obtained results apply to native minority group members (e.g., the Basque people of Spain).

## Conclusions and Policy Implications

Today’s young adults in Europe are living in increasingly culturally and ethnically diverse societies with growing influxes of immigrants and refugees across the national borders. By providing ample opportunities for inter-ethnic interactions, these societal changes have been expected to benefit young generations and make them more positive and tolerant toward newcomers (Korol et al., 2018). Instead, we have seen a rise in anti-immigrant feelings and xenophobic behaviors among native majority youth across European countries (Jones & Rutland, 2018). This points to the urgent need to develop deeper understanding of the individual-level factors and processes that might generate anti-immigrant attitudes among native young adults and how to reduce these negative outgroup orientations. The present longitudinal study makes an important contribution to the literature by examining the relationship between subjective well-being, political distrust, and anti-immigrant attitudes. Its findings indicate that the relationship between subjective well-being and political distrust, on the one hand, and political distrust and anti-immigrant attitudes, on the other, is bidirectional and mutually reinforcing. Moreover, the evidence obtained in the study suggests that adopting anti-immigrant attitudes among native youth might trigger negative perceptions of individual life situation.

Which policy implications can we draw from these results? Our findings suggest that improving young adult’s personal well-being may represent a very important basis for breaking the potential link between low subjective well-being and political distrust, on the one hand, and political distrust and anti-immigrant attitudes, on the other. A large body of existing research on personal well-being has made several policy recommendations on how to enhance youth’s subjective well-being, including implementation of social welfare programs and active labor market policies, building strong social safety nets, cultivation of a positive social atmosphere in working and educational environment, and improving personal and family relationships (for further discussion, see Allen, 2018). Furthermore, prior research (for reviews, see, for example, Allen, 2018; Sin & Lyobomirsky, 2009) has provided substantial evidence that positive psychology interventions (i.e., aimed at cultivating positive cognitions, feelings, or behaviors) serve as effective tools to enhance youth’s personal well-being in a number of domains, including in workplace, educational, and recreational settings. Hence, by applying these policy recommendations and psychological interventions at regional and national levels, we may be able to not only increase young adults’ personal well-being, but also prevent the development of negative perceptions of the broader society (and of the political domain in particular) among these youth. This in turn might potentially reduce their susceptibility to adopt exclusionary attitudes toward immigrant-origin individuals.

## Note

1 The measure of the perceived SES used in the present study assessed young adults’ difficulty in handling their ongoing expenses (for food, rent, other household items, etc.) and, thus, represented an objective SES marker. The correlations between perceived SES and subjective well-being in our study were low: the correlations of perceived SES with subjective well-being (SWL) were −.39, *p* < .001, at T1 and −.33, *p* < .001, at T2; and the correlation of perceived SES at T2 with SWL at T1 was −.32, *p* < .001, and with SWL at T2 was −.39, *p* < .001.
